# Systematic elucidation of the pharmacological mechanisms of Rhynchophylline for treating epilepsy via network pharmacology

**DOI:** 10.1186/s12906-020-03178-x

**Published:** 2021-01-06

**Authors:** Hua Geng, Xuqin Chen, Chengzhong Wang

**Affiliations:** 1 Department of Pediatrics, Maternal and Child Health Hospital of Yancheng, No. 21 Century avenue, Yancheng, 224002 Jiangsu Province China; 2 Pediatric neurology department, Children’s Hospital Affiliated To Suzhou University, No. 50 Donghuan road, Suzhou, 215000 Jiangsu Province China

**Keywords:** Rhynchophylline, Epilepsy, Drug targets, Gene ontology and Kyoto encyclopedia of genes and genomes, Network pharmacology

## Abstract

**Background:**

Epilepsy, one of the most common neurological disorders, affects over 70 million people worldwide. Rhynchophylline displays a wide variety of pharmacologic actives. However, the pharmacologic effects of rhynchophylline and its mechanisms against epilepsy have not been systematically elucidated.

**Methods:**

The oral bioavailability and druglikeness of rhynchophylline were evaluated using the Traditional Chinese Medicine Systems Pharmacology Database. Rhynchophylline target genes to treat epilepsy were identified using PharmMapper, SwissTargetPrediction and DrugBank databases integration. Protein-protein interaction analysis was carried out by utilizing the GeneMANIA database. WebGestalt was employed to perform Gene ontology and Kyoto Encyclopedia of Genes and Genomes pathway enrichment analyses. The drug-disease-target-Gene Ontology-pathway network was constructed using Cytoscape.

**Results:**

The oral bioavailability and druglikeness of rhynchophylline were calculated to be 41.82% and 0.57, respectively. A total of 20 rhynchophylline target genes related to epilepsy were chosen. Among the 20 genes and their interacting genes, 54.00% shared protein domains and 16.61% displayed co-expression characteristics. Gene ontology, Kyoto Encyclopedia of Genes and Genomes and network analyses illustrate that these targets were significantly enriched in regulation of sensory perception, morphine addiction, neuroactive ligand-receptor interaction and other pathways or biological processes.

**Conclusion:**

In short, rhynchophylline targets multiple genes or proteins, biological processes and pathways. It shapes a multiple-layer network that exerts systematic pharmacologic activities on epilepsy.

## Background

Epilepsy is a chronic nervous system disorder that exhibits abnormal brain activities, periods of unusual behavior or seizures, and sensations [1]. It affects over 70 million people of all ages worldwide. About 80% of people with epilepsy live in low or middle income countries. Nearly 70% of people with epilepsy can live seizure free if diagnosed and treated properly. However, over 75% of individuals are untreated, or even stigmatized in poor or developing countries [2].

Traditional Chinese medicine (TCM) contains the most abundant bioactive compounds or pharmaceutic ingredients for drug development. *Uncaria rhynchophylla (U. rhynchophylla)*, for example, is a vital TCM that has been widely utilized in many Asian countries for centuries [3]. It is also known as Gou Teng in China, and found throughout Southern of China, including regions such as, Guangxi, Sicuan, Yunnan, and Fujian [4]. *U. rhynchophylla* belongs to the Rubiaceae family [4] and exerts a wide variety of pharmacologic activities, like anti-inflammatory effects and neurotransmitter regulation [3].

**Fig. 1:**
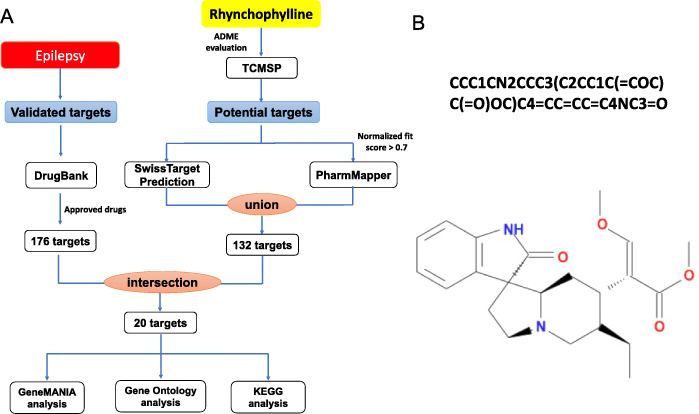
**a** An overview of network pharmacology based approaches for exploring the pharmacologic mechanism of Rhy to treat epilepsy. **b** Canonical SMILES string and chemical structure of Rhy

Attention has been closely paid to *U. rhynchophylla* due to its potential role in the prevention and treatment of a wide variety of diseases, especially for epilepsy [3]. One of the most abundant active pharmacological components of *U. rhynchophylla* is rhynchophylline (Rhy, Fig. 1b), which composes 28–50% of the total alkaloid content in *U. rhynchophylla* [5, 6]. These results indicate Rhy can be utilized to treat epilepsy.

However, the potential molecular functions and involved pathways that Rhy induces have not yet been studied. Network pharmacology provides a novel avenue to elucidate the molecular mechanisms behind TCM through pharmacokinetic evaluation, target prediction and network construction. This is an emerging approach for drug discovery based on poly-pharmacology and systems biology, and has been successfully utilized to unveil the multi-targets and synergistic effects of TCM. For instance, Chen et al. have systematically investigated the mechanisms behind Salvianolic Acid A (SAA) via network pharmacology [7]. Application of network pharmacology approaches to predict the potential drug targets and unveil the latent mechanism will save time and effort. More importantly, a network pharmacology based approach facilitates the progress of drug development. Here, we elucidated systematically the pharmacologic effects of Rhy for the treatment of epilepsy using computational approaches in present study. A workflow of the analysis processes for Rhy targets identification and mechanism investigation is shown in Fig. 1a.

## Methods

### Evaluation of oral bioavailability and drug likeness

The Traditional Chinese Medicine Systems Pharmacology database (TCMSP, http://lsp.nwu.edu.cn/tcmsp.php), is a systematic pharmacology resource [8] that provides the absorption, distribution, metabolism and excretion (ADME) properties of TCMs or compounds, including information on oral bioavailability (OB), druglikeness (DL), and blood-brain barrier (BBB) [9, 10].

Among the provided information, DL and OB are the most important properties of administered drugs, since they aid in assessing the effects of drug distribution in the circulatory system, and how drug like compounds are in relation to factors like bioavailability. In the TCMSP database, DL was evaluated based on molecular descriptors and Tanimoto coefficients, and OB was calculated using OBioavail1.1 based on an in-house model [8, 9].

Here, “rhynchophylline” was put into the TCMSP database and the pharmacokinetics properties of it were evaluated at the molecular level.

### Identification of targets via integrated database analyses

Putative targets of Rhy were predicted by using PharmMapper [11] and SwissTargetPrediction [12], while the validated epilepsy therapeutic targets were collected from the DrugBank [13] database. Overlaps between the predicted targets of Rhy and validated epilepsy therapeutic targets were considered as drug targets of Rhy for treating epilepsy.

PharmMapper (http://www.lilab-ecust.cn/pharmmapper/) is a reverse screening web tool was used to predict putative targets prediction of small molecules [11]. The Mol2 format file of Rhy downloaded from TCMSP was uploaded to the PharmMapper database. Human Protein targets only was selected along with other default arguments. Targets with a normalized fit score greater than 0.7 were selected for further investigation.

SwissTargetPrediction is a web tool used to identify the most possible target genes of small molecules, based on similarity principles via reverse docking [12]. A canonical SMILES string of Rhy was obtained from the PubChem database and uploaded to the SwissTargetPrediction server with all default parameters.

DrugBank is an online cheminformatics and freely accessible database that contains detailed information on drugs with comprehensive drug targets information [13]. The word “epilepsy” was put into DrugBank and only targets of drug approved by the Food and Drug Administration (FDA) acting on epilepsy were collected.

### GeneMANIA analysis

GeneMANIA is an user-friendly and flexible online tool for analyzing gene sets, generating hypotheses concerning gene functions, and prioritizing genes for functional assays [14].

GeneMANIA identifies the most related genes based on correlations of curated genomics and proteomics information. After selecting *Homo sapiens* from the optional organisms, the target genes we identified were entered into the search box, and the results were further collected.

### Gene function and pathway enrichment analyses

The Web-based gene set analysis toolkit (WebGestalt) was employed to explore systematically the functions and pathways of drug targets [15]. The gene list was entered into the WebGestalt web server using the overrepresentation enrichment analysis (ORA) approach with Gene Ontology (GO) and Kyoto Encyclopedia of Genes and Genomes pathway (KEGG) pathway databases and other default parameters. A false discovery rate (FDR) below 0.05 was considered as statistically significant.

GO analysis is extensively employed to annotate gene functions including molecular functions, and cellular components and biological processes, [16]. KEGG is used to systematically investigate pathway information for genes [17].

### Network construction

To comprehensively understand the complex associations among the drug, disease, drug targets and associated biological process/pathways, Cytoscape (v 3.8.2) was employed to construct and analyze the multiple-layer network. Topological analysis was carried out using the network analyzer module in Cytoscape. Three topological properties including closeness, betweenness and degree were applied to evaluate the central properties of the nodes in the network.

## Results

### Pharmacokinetics properties of Rhy

TCMSP provides detailed information for key ADME-related activities like human OB, DL, and BBB. Using the TCMSP, AMDE-related properties of Rhy were deeply and fully studied. Of note, the OB and DL of Rhy were calculated as 41.82% and 0.57, respectively (Fig. 1b and Table 1), which were more higher than the TCMSP recommended thresholds (OB ≥ 30%, DL ≥ 0.18) [8]. These findings indicated that Rhy was chemically suitable for drug development.

**Table 1:** Pharmacological and molecular properties of Rhy

ID	MW	AlogP	Hdom	Hacc	OB(%)	Caco2	BBB	DL	FSAF	HL
Rhy	384.2	1.92	1	6	41.82	0.47	0.38	0.57	0.23	13.2

### Drug targets of Rhy for treating epilepsy

Putative targets of Rhy were predicted using PharmMapper [11] and SwissTargetPrediction [12]. After removing duplicates, 132 putative Rhy targets were obtained by combining information between two databases. A total of 176 validated therapeutic targets for epilepsy were acquired from the DrugBank database (Table S1). To understand the potential mechanisms of Rhy on epilepsy, those two parts targets were intersected and 20 genes in total were obtained finally. These 20 identified target genes were used for further analyses (Table 2).

**Table 2:** Target genes of Rhy for treating epilepsy

No.	Gene ID	Gene Symbol	Target Name
1	134	ADORA1	adenosine A1 receptor
2	153	ADRB1	adrenoceptor beta 1
3	472	ATM	ATM serine/threonine kinase
4	771	CA12	carbonic anhydrase 12
5	760	CA2	carbonic anhydrase 2
6	8913	CACNA1G	calcium voltage-gated channel subunit alpha1 G
7	1137	CHRNA4	cholinergic receptor nicotinic alpha 4 subunit
8	4128	MAOA	monoamine oxidase A
9	4129	MAOB	monoamine oxidase B
10	4985	OPRD1	opioid receptor delta 1
11	4986	OPRK1	opioid receptor kappa 1
12	4988	OPRM1	opioid receptor mu 1
13	10,846	PDE10A	phosphodiesterase 10A
14	5137	PDE1C	phosphodiesterase 1C
15	5138	PDE2A	phosphodiesterase 2A
16	8622	PDE8B	phosphodiesterase 8B
17	5152	PDE9A	phosphodiesterase 9A
18	5290	PIK3CA	phosphatidylinositol-4,5-bisphosphate 3-kinase catalytic subunit alpha
19	6331	SCN5A	sodium voltage-gated channel alpha subunit 5
20	6532	SLC6A4	solute carrier family 6 member 4

### GeneMANIA analysis

**Fig. 2:**
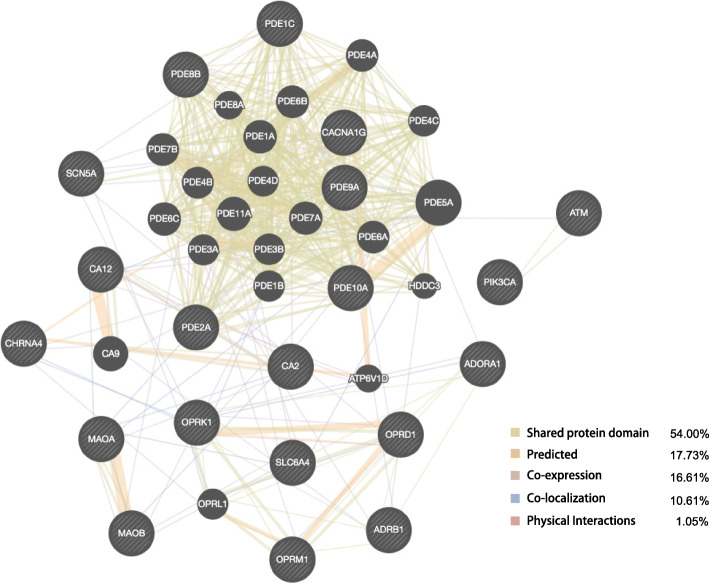
PPI network of the Rhy target genes. Black nodes indicate target genes, while the edge colors represent different interactions. Genes in black circles were the query terms and these in grey circle illustrate genes related to the query genes

Protein-protein interaction (PPI) analysis was carried out using GeneMANIA to investigate the complex functional interaction network. Among the 20 target genes and their interacting genes, 54.00% shared protein domain, 17.73% were predicted, and 16.61% displayed co-expression characteristics. Other results, including co-localization and physical interactions, are demonstrated in Fig. 2. Altogether, these data suggested that Rhy may exert pharmacological functions via affecting multiple genes with shared protein domain or co-expression properties.

### GO and KEGG pathway analyses

**Fig. 3:**
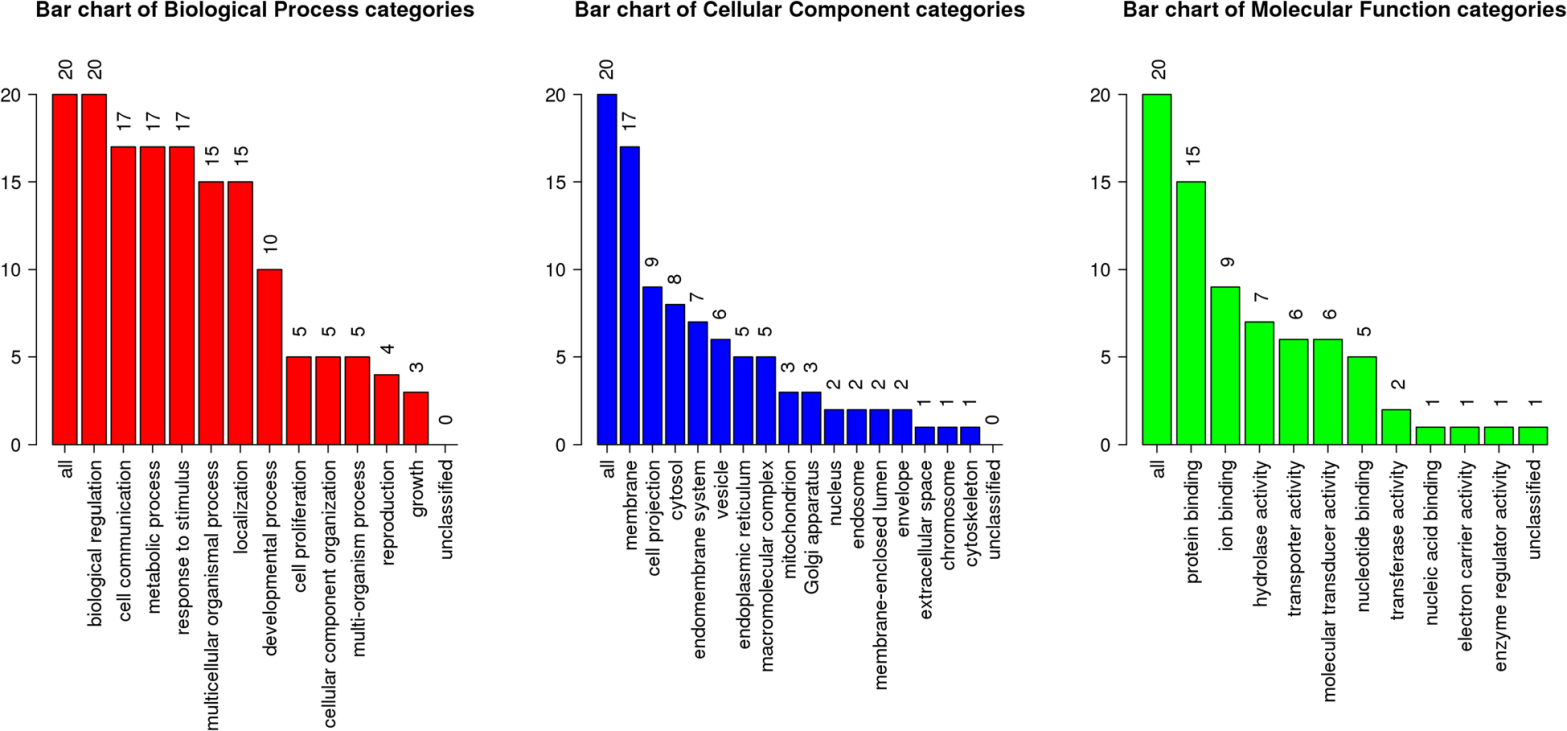
GO map of target genes. **a** Biological process categories. **b** Cellular component categories. **c** Molecular function categories

For further understanding the biological functions and involved pathways of the 20 identified target genes, GO and KEGG enrichment analyses were carried out using WebGestalt. The top seven engaged functions included biological regulation (20/20), cell communication (17/20), metabolic process (17/20), response to stimulus (17/20), and membrane (17/20) (Fig. 3). These GO terms were related to anti-inflammation, in particular anti-inflammation associated with epilepsy.

**Fig. 4:**
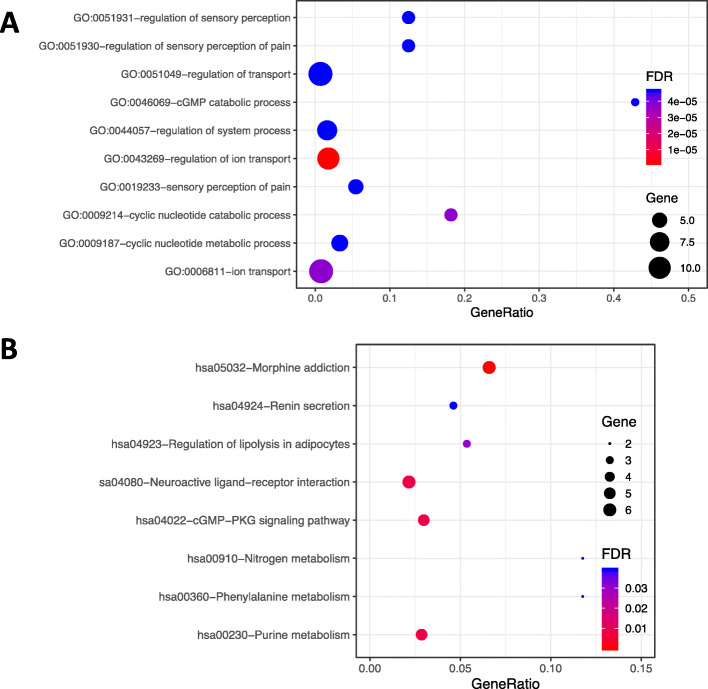
Biological process (**a**) and pathway (**b**) enrichment analyses of target genes. X-axis represents gene ratio (gene count/gene size) while y-axis illustrates the enriched terms with ID and name

Moreover, we found that the 20 target genes were significantly related to regulation of sensory perception, regulation of transport, cGMP catabolic process, regulation of system process and iron transport, etc. (Fig. 4a and Table S2). As for the pathway analysis, genes enriched in the 8 KEGG pathways with significant FDRs included genes involved in morphine addiction, neuroactive ligand-receptor interaction, renin secretion, and the cGMP-PKG signaling pathway (Fig. 4b and Table S2).

### Network analysis

**Fig. 5:**
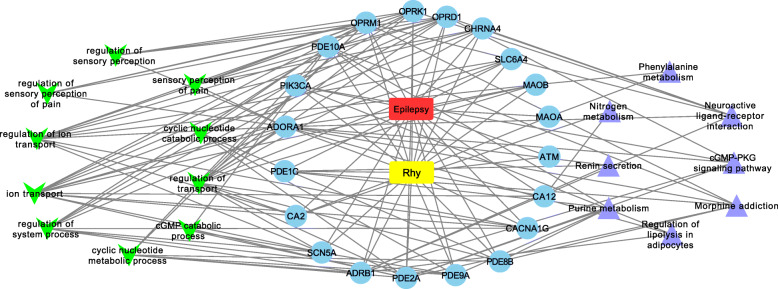
Drug-disease-target-GO-pathway network. The yellow and red oblong, blue circles, green inverted triangles, and purple triangles represent rhynchophylline, epilepsy, target genes and related GO biological processes and KEGG pathways, respectively

Based on target identification, GO and pathway enrichment analyses, an entire drug, disease, targets and involved GO terms and KEGG pathways network was constructed using Cytoscape (v 3.8.2). This five-layer network contained 40 nodes and 141 edges (Fig. 5), suggesting multiple targets and multiple effects characteristics of Rhy for treating epilepsy,. Among these nodes, 20 were target gene nodes, 10 were GO function nodes and 8 were KEGG pathway nodes. The yellow and red oblong, blue circles, green inverted triangles, and purple triangles represent rhynchophylline, epilepsy, target genes and related GO biological processes and KEGG pathways, respectively. The degree of the 20 bioactive target genes in the network were displayed in Table 3. Among these target genes, 5 had a high degree (degree ≥10), these included ADOR1 (degree = 14), OPRM1 (degree = 12), OPRD1 (degree = 11), PDE2A (degree = 10) and OPRK1 (degree = 10).

**Table 3:** The topological parameters of target genes sorted by degree

Target genes	Degree	Closeness	Betweenness
ADORA1	14	0.557	0.119
OPRM1	12	0.527	0.069
OPRD1	11	0.513	0.051
PDE2A	10	0.5	0.075
OPRK1	10	0.5	0.039
ADRB1	8	0.476	0.043
PDE9A	7	0.464	0.038
PDE10A	7	0.464	0.033
PDE8B	7	0.464	0.029
CHRNA4	7	0.464	0.016
MAOB	6	0.453	0.037
CA2	6	0.453	0.034
CACNA1G	6	0.453	0.008
SCN5A	6	0.453	0.008
PDE1C	5	0.443	0.019
SLC6A4	5	0.443	0.004
CA12	4	0.433	0.021
PIK3CA	4	0.433	0.011
MAOA	3	0.424	0.017
ATM	2	0.415	0

## Discussion

Epilepsy, one of the most common serious brain diseases, affects 70 million people worldwide [2]. However, 30–40% of epileptic patients are resistant to western medicine and affected by side effects of these drugs [18]. TCM or natural products have been utilized widely to prevent and treat numerous diseases due to their high efficiency, small side effects and lack of drug resistance [19, 20]. Therefore, the development of active compounds extracted from TCM on drug design and discovery process should be prioritized urgently [21–24]. Here, we performed network pharmacology based analyses to screen the putative active compound and hidden mechanism of *U. rhynchophylla* acting on epilepsy.

Based on ADME properties, Rhy (OB = 41.82%, DL = 0.57) was screened as a potential bioactive compound for drug development. A total of 20 candidate targets of Rhy against epilepsy were identified, reflecting the multiple targets paradigm of TCM,. GeneMANIA analysis suggested that these target genes and their associated genes share the same protein domain and have identical or similar functions.

To investigate the synergistic mechanisms of Rhy for the treatment of epilepsy, we carried out GO and KEGG pathway enrichment analysis. We identified an anti-inflammatory role of Rhy acting on epilepsy. Hsu et al. reported that seizures result in the inflammation of the central nervous system [25]. Moreover, we found that Rhy targets were closely related to biological processes, such as regulation of sensory perception, cGMP catabolic process, and regulation of system process. These findings were confirmed by previous experimental studies or reviews. For example, Campen et al. reported altered sensory sensitivity was associated with epilepsy susceptibility in childhood [26]. Nieoczym et al. revealed the involvement of the cGMP pathway in seizure processes [27]. Sedigh-Sarvestani et al. stated that regulatory systems, including the consciousness system and autonomic nervous system, play a vital role in the epileptic networks [28].

In addition, we discovered that the 20 Rhy targets acting on epilepsy were significantly enriched in morphine addiction, neuroactive ligand-receptor interaction, cGMP-PKG signaling and other pathways. Saboory et al. studied the mechanisms of morphine enhancement of spontaneous seizure activity and found that morphine at a low concentration (10 microM) depresses seizure activity [29]. Krasniqi et al. recently clarified the mechanism of renin in brain induced neuropathology like epilepsy, and discovered renin inhibitors may have therapeutic applications for epilepsy treatment [30]. Liu et al. reported that abnormal neuroactive ligand-receptor interactions enhance the susceptibility to epileptic seizures [31]. Moreover, Risley et al. proposed that the cGMP-PKG signaling pathway was a hidden new target for studying epilepsy mechanisms [32]. These finding closely coincide with the results obtained through GO and KEGG analyses.

The multiple-layer network illustrated that Rhy has multiple targets to exert multiple pharmacologic effects, reflecting multiple targets, multiple effects and complex disease. In total, 5 of 20 targets displayed a degree larger than 10 in the network, including ADOR1, OPRM1, OPRD1, PDE2A and OPRK1. Some of them have been verified by animal experiments or clinical trials. Kasai et al. reported that the OPRM1 polymorphism was associated with epilepsy susceptibility [33]. Similarly, Doummar et al. also reported PDE2A variants, identified by whole-exome sequencing, caused cognitive impairment and occurred in epilepsy patients [34]. Ueda et al. revealed an upregulation of OPRK1 in a rat model of posttraumatic epilepsy [35]. There are no previous studies directly reporting associations between the other 2 key targets (ADOR1, OPRD1) and epilepsy.

Taken together, we propose that Rhy may be a promising compound that can be applied for further drug development in the treatment of epilepsy. Despite these findings, there were still some bias and limitations in this study owing to the databases utilized in analyses. Further studies are necessary to validate the acting mechanisms via pharmacokinetic tests.

## Conclusion

In conclusion, Rhy is a promising and valuable compound for the development of a safe and effective multi-targeted anti-epilepsy therapy. This study sheds insight on the perspectives and challenges of Rhy research and clinical applications acting on epilepsy in the future.

## Supplementary Information

**Additional file 1.** Detail information of the putative targets of Rhy and validated therapeutic targets on epilepsy.

**Additional file 2.** Detail information of GO and KEGG enrichment analyses.

## Data Availability

All main data has been presented in the form of tables in additional file. Other data used to support the findings of this study are available from the corresponding author upon request.

## Abbreviations

TCMSP: Traditional Chinese Medicine Systems Pharmacology Database
GO: Gene Ontology
TCM: Traditional Chinese Medicine
*U. rhynchophylla*: *Uncaria rhynchophylla*
Rhy: rhynchophylline
ADME: Absorption, Distribution, Metabolism and Excretion
DL: Drug Likeness
OB: Oral Bioavailability
BBB: Blood-Brain Barrier
FDA: Food and Drug Administration
PPI: Protein-protein interaction
WebGestalt: Web-based Gene set analysis toolkit
ORA: Overrepresentation Enrichment Analysis
FDR: False discovery rate
KEGG: Kyoto Encyclopedia of Genes and Genomes pathway
